# Hydrogen sulfide plays an important role by regulating endoplasmic reticulum stress in myocardial diseases

**DOI:** 10.3389/fphar.2023.1172147

**Published:** 2023-04-13

**Authors:** Huijie Zhao, Xiaodi Fu, Yanting Zhang, Yihan Yang, Honggang Wang

**Affiliations:** ^1^ Institute of Chronic Disease Risks Assessment, Henan University, Kaifeng, China; ^2^ School of Basic Medical Sciences, Henan University, Kaifeng, Henan, China; ^3^ School of Clinical Medicine, Henan University, Kaifeng, Henan, China

**Keywords:** endoplasmic reticulum stress, hydrogen sulfide, myocardial diseases, oxidative stress, apoptosis

## Abstract

Endoplasmic reticulum (ER) is an important organelle for protein translation, folding and translocation, as well as the post-translational modification and assembly of newly synthesized secreted proteins. When the excessive accumulation of misfolded and/or unfolded proteins exceeds the processing capacity of ER, ER stress is triggered. The integrated intracellular signal cascade, namely the unfolded protein response, is induced to avoid ER stress. ER stress is involved in many pathological and physiological processes including myocardial diseases. For a long time, hydrogen sulfide (H_2_S) has been considered as a toxic gas with the smell of rotten eggs. However, more and more evidences indicate that H_2_S is an important gas signal molecule after nitric oxide and carbon monoxide, and regulates a variety of physiological and pathological processes in mammals. In recent years, increasing studies have focused on the regulatory effects of H_2_S on ER stress in myocardial diseases, however, the mechanism is not very clear. Therefore, this review focuses on the role of H_2_S regulation of ER stress in myocardial diseases, and deeply analyzes the relevant mechanisms so as to lay the foundation for the future researches.

## 1 Introduction

### 1.1 Overview of endoplasmic reticulum stress

**FIGURE 1:**
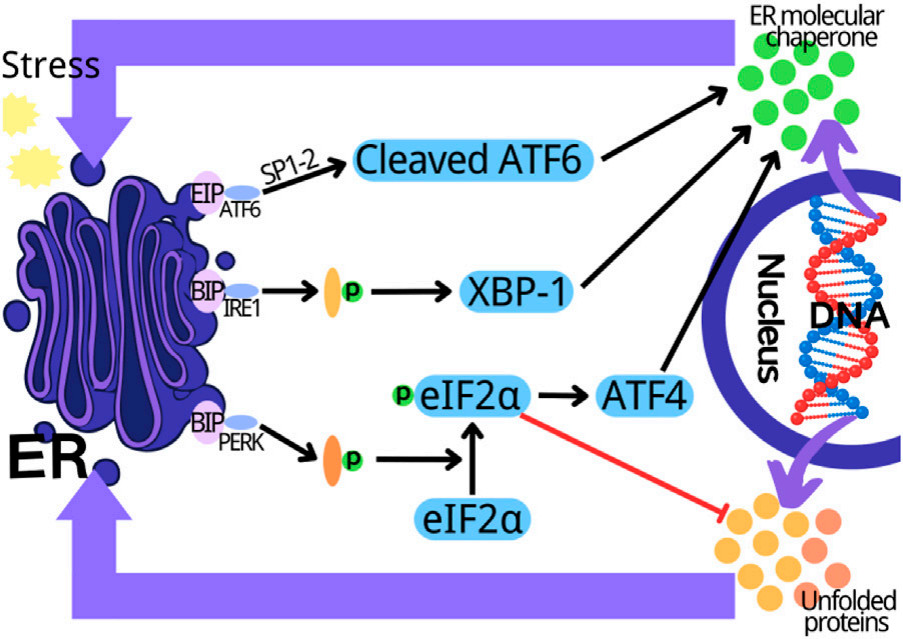
Three parallel signal transduction pathways in endoplasmic reticulum stress. PERK, pancreatic endoplasmic reticulum kinase; IRE1, inositol dependent enzyme 1; ATF6, activate transcription factor 6; XBP1, X-box binding protein 1; ER, endoplasmic reticulum.

Endoplasmic reticulum (ER) is an organelle of eukaryotic cells, which is responsible for the synthesis of proteins, carbohydrates and lipids, and regulates the intracellular calcium concentration via the storage and release of calcium (Wu et al., 2018). In ER cavity, the newly synthesized peptides are folded and modified to ensure their accurate conformation and function. The dysfunction of this process induces the accumulation of misfolded or unfolded proteins in ER, thus triggering the unfolded protein response (UPR) to result in ER stress (Byrd and Brewer, 2012). UPR can restore ER homeostasis, however, if it fails, UPR will trigger cell death (Almanza et al., 2019). A variety of physiological and pathological factors can induce ER stress, including ER oxidative stress, nutritional deficiency, abnormal calcium content, lipid overload, iron imbalance, hypoxia, cancer and infection (Kaufman et al., 2002; Marciniak and Ron, 2006; Martins et al., 2016). The ER stress/UPR is mediated by three parallel signal pathways: the activated transcription factor 6 (ATF6) mediated pathway; the pancreatic endoplasmic reticulum kinase (PERK) mediated pathway and the inositol dependent enzyme 1 (IRE1) mediated pathway (Chen et al., 2022; Lu et al., 2022). When there is no external stimulation, the binding immunoglobulin (BIP) combines with PERK, IRE1 and ATF6 to inhibit their activation. The external stimulation and unfolded/misfolded proteins promote the separation of BIP from PERK, ATF6 and IRE, thus activating them. Subsequently, the self-phosphorylated PERK inhibits the protein synthesis and increases ATF4 expression by phosphorylating eIF2a. The protease 1 (SP1) and protease 2 (SP2) in Golgi complex cut the separated ATF6, and the self-phosphorylated IRE1 cleaves XBP1 mRNA. The cut XBP1, ATF4, and ATF6 upregulate the expression of ER chaperone gene, and further participate in the elimination of the unfolded and misfolded protein in ER, and the recovery of normal cell homeostasis (Figure 1) (Wang et al., 2020; Lv et al., 2021a; Zhao et al., 2021b; Zhao et al., 2022a). ER stress plays a key role in many types of physiological and pathological processes, including cancer, diabetes, neurodegeneration, inflammation and fibrosis, as well as the physiological events related to organ function and development (Harada et al., 2021). In recent years, the increasing evidences indicate that ER stress is involved in myocardial diseases, however, the related mechanism is not fully understood.

### 1.2 Overview of hydrogen sulfide

Hydrogen sulfide (H_2_S) is a flammable, volatile and colorless gas with a smell similar to rotten eggs (Arif et al., 2021). Since Bernardino Ramazzini firstly described hydrogen sulfide (H_2_S) as a toxic gas in 1713, many studies on H_2_S have focused on its toxicity (Reiffenstein et al., 1992). In 1989, Warnycia et al. confirmed the existence of endogenous H_2_S in brain, which indicated that H_2_S may have the physiological effects (Warenycia et al., 1989). Since then, the researches have been mainly carried out to reveal its multiple regulatory functions (Dilek et al., 2020). In recent years, with the deepening of researches on H_2_S, H_2_S has been considered to be the third gas signal molecule with multiple biological functions after nitric oxide (NO) and carbon monoxide (CO) (Paul and Snyder, 2018; Zaorska et al., 2020). In mammalian organisms, H_2_S can be produced through the non-enzymatic and enzymatic pathways. The non-enzymatic process is mainly produced by the decomposition of the inorganic substances, which has a very small contribution to the production of H_2_S (Xiao et al., 2021). Cystathionine-γ-lyase (CSE), cystathionine-β-synthase (CBS) and 3-mercaptopyruvate sulfurtransferase (3-MST) are the three important enzymes of the mammalian enzymatic pathway to produce H_2_S (Luo et al., 2020). The distribution of the three enzymes that catalyze the endogenous H_2_S production has tissue and system specificity. CSE is mainly distributed in the cardiovascular system. Recent studies have shown that it is also expressed in kidney, lung and liver. CBS is the main H_2_S synthetase in the central nervous system, kidney and liver. 3-MST is widely expressed in liver, kidney, lung and vascular system (Wang, 2012; Lv et al., 2021b). During the enzymatic pathway of H_2_S production, CBS catalyzes the β substitution reaction of homocysteine and serine, thus generating L-cystathionine. CSE acts as a catalyst for L-cystathionine to eliminate α, γ -cysteine, and in turn to generate L-cystenine. Next, CSE/CBS catalyzes L-cystenine for the β elimination reaction to generate H_2_S. Meanwhile, cysteine aminotransferase (CAT) also catalyzes L-cystenine, which transfers the amine to α -ketoglutarate to generate 3-mercaptopyruvate (3-MP). The 3-MP is then catalyzed as H_2_S by 3-MST (Lv et al., 2021b; Zhao et al., 2021b; Zhao et al., 2022b). The increasing evidence indicates that H_2_S participates in many kinds of pathological and physiological processes, including anti-inflammation (Zhao et al., 2019), anti-apoptosis (Li et al., 2019), vasodilation (Greaney et al., 2017; Jin et al., 2017), anti-oxidative stress (Tocmo and Parkin, 2019), cell differentiation, cell proliferation/hypertrophy and cell survival/death (Zhang et al., 2017a) (Figure 1). Therefore, H_2_S plays an important role in multiple diseases by regulating ER stress (Ge et al., 2019; Chen et al., 2021b), including myocardial diseases. However, the relevant mechanism is not fully understood. In this review, we focused on the progresses about the H_2_S regulation of ER stress in myocardial diseases and deeply analyzed the relevant mechanisms to provide the foundation for the future researches.

## 2 Hydrogen sulfide plays an important role by regulating endoplasmic reticulum stress in diabetes cardiomyopathy

### 2.1 Exogenous hydrogen sulfide improves diabetes cardiomyopathy through reactive oxygen species (ROS)/endoplasmic reticulum stress/autophagy/apoptosis pathway

Diabetes cardiomyopathy (DCM) is a pathophysiological condition caused by diabetes, which induces heart failure without hypertension, coronary artery diseases and valvular heart diseases (Dillmann, 2019; Lorenzo-Almorós et al., 2022; Nakamura et al., 2022). Although there have been many studies on DCM in recent years, its pathogenesis still needs to be further clarified (Zhao et al., 2022c; Jia et al., 2022). The evidence indicates that DCM is related to oxidative stress and apoptosis (Thandavarayan et al., 2011; Kumar et al., 2013). Rui Yang and colleagues used the method of intraperitoneal injection of streptozotocin to prepare the rat model of diabetes. The results showed that in diabetes rats the left ventricular function and the activity of superoxide dismutase (SOD) and glutathione peroxidase (GSH-Px) were significantly decreased, the myocardial structure was notably damaged, the content of malondialdehyde (MDA) in myocardial tissue was increased, and the mRNA expressions of GRP78, CHOP and caspase 12 were significantly upregulated, which were reversed by NaHS (a donor of H_2_S). It could be deduced that exogenous H_2_S protected myocardial injury by reducing oxidative stress injury and inhibiting ER stress, which needed to be futher confirmed (Yang et al., 2016). Reactive oxygen species (ROS) are the main substances that induce oxidative stress in body (Tu et al., 2021). Moreover, ROS can induce ER stress (Feng et al., 2022), and H_2_S can improve cardiomyopathy by eliminating ROS (Zhang et al., 2017b). Therefore, it can be deduced that exogenous H_2_S can inhibit ER stress through reducing ROS/oxidative stress to improve DCM (Yang et al., 2016). Another study by FANG LI et al. confirmed the above conclusion that exogenous H_2_S improved DCM by inhibiting ER stress. The results revealed that exogenous H_2_S had no effects on body weight (BW), heart weight (HW), the ratio of HW/BW and the blood glucose concentration of STZ-induced DCM rat model. In STZ-induced DCM rat model, hyperglycemia led to myocardial fibrosis evidenced by the loose and disordered myocardial tissue, and induced myocardial collagen fibrosis evidenced by the significantly increased collagen with tissue disorder, which were reversed by NaHS. Moreover, NaHS downregulated ER stress induced by hyperglycemia through decreasing the expression levels of ERS markers caspase-12, GRP-78 and CHOP, indicating that exogenous H_2_S might ameliorate DCM through suppressing ER stress (Li et al., 2016). It has been reported that the myocardial apoptosis is upregulated in STZ-induced DCM rat model and plays an important role in DCM (Yu et al., 2014; Zhenzhong et al., 2015). Furthermore, cardiomyocyte apoptosis is closely related with ER stress (Zhang et al., 2020; Ren et al., 2022). Therefore, it can be deduced that exogenous H_2_S may inhibit myocardial apoptosis through suppressing ER stress in DCM (Li et al., 2016). The research by Fan Yang and colleagues is consistent with the above deduction. Their results showed that NaHS mitigated the mitochondrial swelling and the cardiomyocyte apoptosis in rats with DCM. The *in vitro* experiments showed that GYY4137 (a donor of H_2_S) significantly reduced hyperglycemia-induced intracellular ROS level, and NAC (ROS scavenger) attenuated hyperglycemia-induced cardiomyocyte apoptosis, indicating that exogenous H_2_S inhibited HG-induced cardiomyocyte apoptosis by reducing ROS production in DCM. Further experiments showed that GYY4137 and NAC treatment significantly reduced the expression levels of ER stress marker proteins induced by HG in myocardial cells, suggesting that exogenous H_2_S inhibited ER stress in cardiomyocyte of DCM through decreasing ROS production. Collectively, exogenous H_2_S improved DCM by inhibiting cardiomyocyte apoptosis and ER stress through decreasing ROS level (Yang et al., 2017a). The evidence indicates that exogenous H_2_S protects against DCM via the regulation of autophagy (Yang et al., 2017b; Wu et al., 2017), and autophagy and ER stress are involved in DCM (Pei et al., 2018). Furthermore, ER stress regulates autophagy (Wang and Tang, 2020; Zhao et al., 2021a) which in turn regulates apoptosis in DCM (Wu et al., 2020). Therefore, it can be inferred from the above that exogenous H_2_S improves DCM through ROS/ER stress/autophagy/apoptosis pathway (Yang et al., 2017a), which needs to be further confirmed. Maojun Liu and co workers obtained the similar results as above, and proved that the inhibition of janus kinase/signal transducer and activator of transcription (JAK/STAT) signaling pathway is involved in H_2_S inhibition of oxidative stress and ER stress in improvement of DCM (Liu et al., 2018). The signaling pathways involved in H_2_S improvement of DCM through regulating ER stress need to further studied.

**FIGURE 2:**
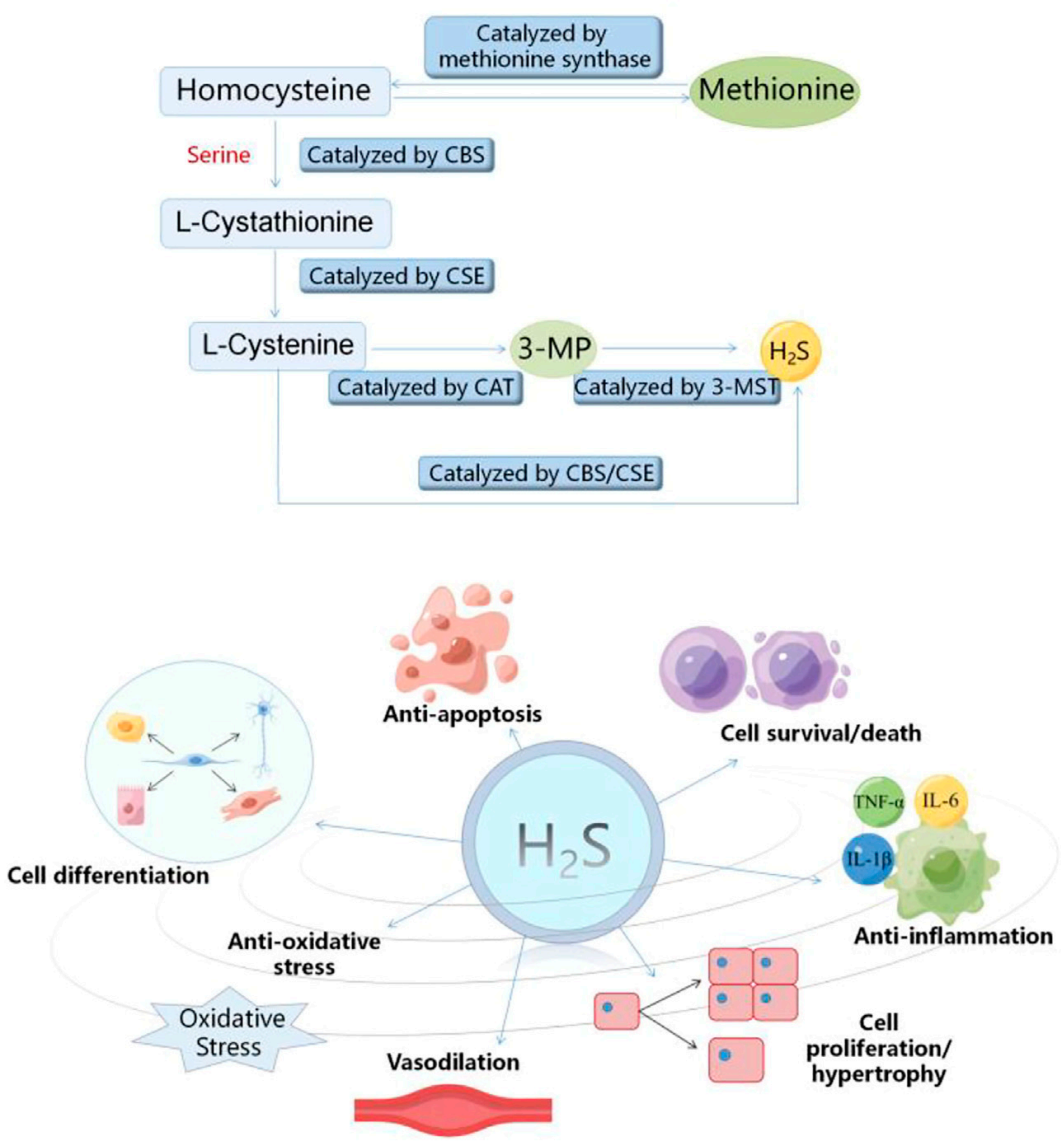
Diagram of endogenous hydrogen sulfide production process and its biological effects. CSE, cystathionine-γ-lyase; CBS, cystathionine-β-synthase; 3-MST, 3-mercaptopyruvate sulfurtransferase.

**FIGURE 3:**
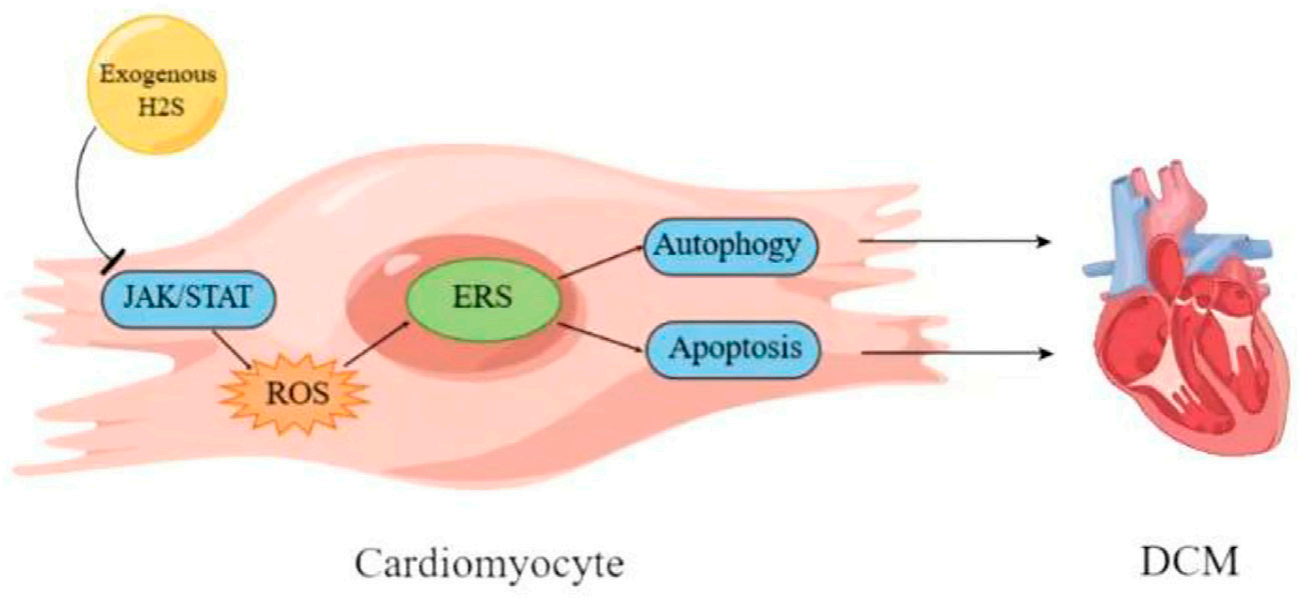
Schematic diagram of mechanism of exogenous H_2_S improving diabetes cardiomyopathy. DCM, diabetes cardiomyopathy; ERS, endoplasmic reticulum stress; JAK/STAT, janus kinase/signal transducer and activator of transcription.

Collectively, exogenous H_2_S may ameliorate DCM through ROS/ER stress/autophagy/apoptosis by inhibiting JAK/STAT signaling pathway (Figures 2, 3).

### 2.2 Endogenous hydrogen sulfide/exogenous hydrogen sulfide regulates endoplasmic reticulum stress in diabetes cardiomyopathy

In addition to exogenous H_2_S, endogenous H_2_S also participates in DCM by regulating ER stress. Runmin Guo and colleagues found that the level of H_2_S in the serum of DCM patients and DCM rats, and the levels of H_2_S and CSE protein in the heart tissue of DCM rats decreased significantly. The level of H_2_S in the supernatant of cardiomyocytes, the cell viability and the lipid deposition of cardiomyocytes induced by palmitic acid (PA) also decreased significantly. The above indicated that deficiency of endogenous H_2_S participated in diabetes-induced myocardial injury. The in-depth research demonstrated that NaHS downregulated ER stress of myocardial cells. Furthermore, the treatment of diabetes rats with NaHS or 4-PBA (an inhibitor of ER stress) alleviated heart lipotoxicity evidenced by the decrease of lipid accumulation, TUNEL positive cells and the cleaved caspase-3 expression, indicating that ER stress participated in DCM. Collectively, endogenous H_2_S was involved in DCM, and exogenous H_2_S ameliorated DCM by inhibiting ER stress (Guo et al., 2017). Whether the mechanism of endogenous H_2_S regulating ER stress is the same as that of exogenous H_2_S remains to be clarified. In addition, our previous studies showed that exogenous H_2_S inhibited the lipid toxicity damage of hepatocytes mediated by oleic acid-induced NLRP3 inflammasome by upregulating autophagy (Wang et al., 2019a). Therefore, whether autophagy and NLRP3 inflammasome participate in H_2_S inhibition of ER stress in improving myocardial lipotoxic injury remains to be clarified.

## 3 Hydrogen sulfide plays an important role by regulating endoplasmic reticulum stress in ischemic/hypoxic myocardial disease

Chronic intermittent hypoxia (CIH) is the main characteristic of the obstructive sleep apnea and an important risk factor of myocardial diseases (Zhu et al., 2020; Hu et al., 2021). CIH induces myocardial injury mainly by promoting oxidative stress and inflammation (Wang et al., 2021). In order to study the influence of DL-propargylglycine (PAG, an inhibitor of endogenous H_2_S production) on CIH-induced myocardial injury, Xiufang Zhou et al. established a rat model of CIH and found that PAG significantly alleviated CIH-induced myocardial injury by increasing left ventricular fractional shortening (LVFS) and left ventricular ejection fraction (LVEF), and decreasing left ventricular end-systolic dimension (LVDs), left ventricular end-diastolic dimension (LVDd), left ventricular end-systolic volvume (LVESV) and left ventricular end-diastolic volume (LVEDV). Moreover, PAG also improved the pathological changes of myocardium (disordered arrangement of cardiomyocyte and broken myocardial fibers) induced by CIH. The cooperation of PAG and CIH significantly reduced the expression of *CSE* gene in myocardial tissue and the level of H_2_S in serum and myocardial tissue. Furthermore, PAG alleviated the increase of the levels of apoptosis index (AI), cleaved caspase-3, and Bax, and the decrease of Bcl-2 level induced by CIH, indicating that PAG inhibited CIH-induced cardiomyocyte apoptosis. In addition, PAG inhibited CIH-induced oxidative stress by decreasing the levels of LPO and ROS and increasing SOD level in cardiomyocytes. CIH increased the expression of ER stress marker proteins in cardiomyocyte, which was reversed by PAG, indicating that PAG alleviated ER stress induced by CIH. These results suggested that PAG has protective effects on CIH-induced myocardial injury by reducing myocardial apoptosis, oxidative stress and ER stress. PAG is an inhibitor of endogenous H_2_S production, implying that endogenous H_2_S is involved in the protection of CIH-induced myocardial injury by reducing myocardial apoptosis, oxidative stress and ER stress (Zhou et al., 2018). In the above study, PAG provides cardiac protection in rats with CIH, but has the opposite effect in the control group. The mechanism needs in-depth studies to clarify. Further, PAG alleviates CIH-induced oxidative stress and ER stress, which contradicts the previous studies that exogenous H_2_S alleviates oxidative stress induced by myocardial ischemia/reperfusion (I/R) (Li et al., 2015; Lv et al., 2021c). The reasons for contradiction need to be further studied.

In addition to hypoxic cardiomyopathy, H_2_S regulation of ER stress also plays an improving role in ischemic cardiomyopathy. Myocardial I/R injury is a serious injury to the ischemic myocardium after blood flow recovery. At present, there is an urgent need to find an effective way to treat myocardial I/R injury clinically (Zheng et al., 2021; Dong et al., 2022). Increasing evidences indicate that miRNAs play an important role in myocardial I/R injury (Zhang et al., 2021; Bei et al., 2022). However, the relevant mechanism is not completely clear. In order to study the role and mechanism of miR-133a in myocardial I/R injury, Lin Ren and colleagues established *in vivo* and *in vitro* models of myocardial I/R injury and found that exogenous H_2_S reduced I/R-induced cardiomyocyte apoptosis and ER stress. Meanwhile, the expression level of miR-133a in cardiomyocyte was significantly inhibited by I/R, which was reversed by exogenous H_2_S. Further, the co-treatment of H_2_S and the overexpression of miR-133a had stronger inhibitory effects on ER stress and the apoptosis of cardiomyocytes induced by hypoxia-reoxygenation (H/R) than that of the treatment of H_2_S or miR-133a overexpression. However, the co-treatment of H_2_S and miR-133a inhibitor had the opposite effects. These indicated that exogenous H_2_S inhibited H/R-induced ER stress and apoptosis of cardiomyocytes through upregulating miR-133a expression. In addition, H/R inhibited the proliferation, migration and invasion of cardiomyocytes, which was reversed by overexpression of miR-133a and H_2_S treatment. While the co-treatment of H_2_S and miR-133a inhibitor had the opposite effects. The similar results were obtained *in vivo* experiments. Collectively, exogenous H_2_S improved myocardial I/R injury through inhibiting I/R-induced ER stress and apoptosis of cardiomyocytes. and enhancing the proliferation, migration, and invasion of cardiomyocytes inhibited by I/R by upregulating miR-133a expression (Ren et al., 2019). The evidence indicates that miR-133a relieves oxidative stress (Guo et al., 2021). In the above study, whether miR-133a can inhibit ER stress by inhibiting oxidative stress remains to be confirmed. The mechanism of H_2_S inhibiting ER stress through miR-133a in MIRI remains to be clarified.

## 4 Hydrogen sulfide plays an important role by regulating endoplasmic reticulum stress in acute myocardial infarction

Acute myocardial infarction (AMI) refers to the acute myocardial injury found in the clinical environment of myocardial ischemia (Occhipinti et al., 2021; Badat and Rangiah, 2022). Myocardial reconstruction often occurs after myocardial infarction (Castelvecchio et al., 2019). The evidence indicates that apoptosis, oxidative stress, ER stress and autophagy are involved in myocardial remodeling after AMI (Tabas, 2009; Park et al., 2017). However, the specific mechanism is not fully clear. In order to investigate the relationship among the myocardial remodeling after AMI, ER stress, autophagy and the induced apoptosis, Yaling Li and colleagues established a rat model of AMI (intraperitoneal injection of high-dose isoproterenol Iso) and the cobalt chloride (CoCl2) induced hypoxia model of H9c2 myocardial cells. The results showed that in the rat model of AMI, the cardiac function was significantly reduced, the myocardial cells were disordered, the myocardial type III collagen, interstitial collagen fibers, TGF-β and other fibrosis-related factors in myocardial tissue was significantly increased, which were reversed by NaHS treatment. In the myocardial tissue of rats with AMI, the expressions of caspase-3, caspase-9 and Bax increased significantly, while Bcl-2 expression decreased notably, which were reversed by NaHS treatment. These results indicated that H_2_S could improve the cardiac function, suppress myocardial reconstruction, and reduce myocardial cell apoptosis in rats with AMI. The mechanism study showed that in rats with AMI, the ER stress-related proteins (BIP/GRP78, CHOP and EIF2α) and the expression of autophagy-related proteins (ATG5, Beclin and ATG16L1) were significantly increased, and the PI3K/AKT signal pathway was inhibited, which were reversed by NaHS treatment. *In vitro*, the agonist of ER stress or autophagy reversed the inhibitory effect of H_2_S on apoptosis of AMI cell model, indicating that the inhibition of cardiomyocyte apoptosis by exogenous H_2_S may be related to the inhibition of ER stress and autophagy. In conclusion, exogenous H_2_S inhibited cardiomyocyte apoptosis by regulating ER stress-autophagy axis, thus improving myocardial remodeling after AMI (Li et al., 2020). This was consistent with the previous conclusion that the excessive cardiomyocyte apoptosis facilitates the deposition of extracellular matrix to promote myocardial fibrosis, thus inducing myocardial reconstruction (Shiraishi et al., 2022), and the excessive autophagy can induce cardiomyocyte apoptosis (Liang et al., 2022). Because the level of ER stress/autophagy is different in different types of cells and different pathological processes, sometimes H_2_S can inhibit ER stress/autophagy, sometimes it is the opposite (Wang et al., 2019b; Wang et al., 2020), so whether H_2_S can promote ER stress/autophagy in AMI remains to be studied. In addition, the detailed mechanism of H_2_S regulating ER stress/autophagy in AMI needs further study.

## 5 Hydrogen sulfide plays an important role by regulating endoplasmic reticulum stress in sepsis-induced myocardial dysfunction

Myocardial dysfunction, including systolic and diastolic dysfunction, is one of the main symptoms of septicemia. However, the underlying mechanism of sepsis-induced myocardial dysfunction (SIMD) remains unclear (Yang et al., 2019; Lin et al., 2020). In order to study the role of H_2_S and ER stress in SIMD, Yu-hong Chen and colleagues used lipopolysaccharide (LPS) to construct SIMD model. The results demonstrated that LPS-treated mice showed characteristic manifestations of the acute myocardial injury and inflammation, including tissue edema, inflammatory cell infiltration and nuclear swelling, and the decrease of the expressions of CBS, CSE and 3-MST. Moreover, CSE KO aggravated the above myocardial lesions, suggesting that the lack of endogenous H_2_S enhanced LPS-induced myocardial dysfunction. The intraperitoneal injection of NaSH in LPS-treated mice significantly improved myocardial function, decreased the plasma levels of cardiac troponin I (cTnI) and creatine kinase (CK), and alleviated the myocardial cell damage and inflammation. In addition, for the mice treated by LPS and CSE KO, the higher concentration of NaSH was required to obtain the similar results, indicating that endogenous H_2_S deficiency contributed to SIMD, and exogenous H_2_S supplementation alleviated LPS-induced myocardial dysfunction. The mechanism research showed that LPS increased the levels of tumor necrosis factor-α (TNF-α), IL-1β and toll-like receptor 4 (TLR4) in the heart and plasma, and the expressions of ER stress marker proteins in heart, which were reversed by NaSH, indicating that H2S reduced the level of inflammation and ER stress (Chen et al., 2021b). It has been reported that the inflammation and ER stress can be regulated *via* TLR4 pathway (Chen et al., 2021a; Xie et al., 2023). Therefore, it can be deduced that exogenous H_2_S suppresses inflammation and ER stress by inhibiting TLR4 pathway, thus improving LPS-induced SIMD (Chen et al., 2021b). These findings revealed the potential role of H_2_S in regulating ER stress in the treatment of SIMD.

## 6 Conclusion

Increasing evidence indicates that H_2_S participates in myocardial diseases by regulating ER stress. In this review, we summrized as follows: 1) exogenous H_2_S improves DCM by inhibiting ER stress through reducing ROS/oxidative stress; 2) exogenous H_2_S may inhibit myocardial apoptosis through suppressing ER stress in DCM; 3) exogenous H_2_S may ameliorate DCM through ROS/ER stress/autophagy/apoptosis by inhibiting JAK/STAT signaling pathway; 4) endogenous H_2_S is involved in DCM, and exogenous H_2_S ameliorates DCM by inhibiting ER stress; 5) endogenous H_2_S protects against CIH-induced myocardial injury by reducing myocardial apoptosis, oxidative stress and ER stress; 6) exogenous H_2_S improves myocardial I/R injury through inhibiting I/R-induced ER stress and apoptosis of cardiomyocyte. and enhancing the proliferation, migration, and invasion of cardiomyocytes inhibited by I/R by upregulating miR-133a expression; 7) exogenous H_2_S ameliorates myocardial remodeling after AMI by suppresseing cardiomyocyte apoptosis through regulating ER stress-autophagy axis; 5) endogenous H_2_S deficiency contributes to SIMD, and exogenous H2S improves LPS-induced SIMD through suppressing inflammation and ER stress by inhibiting TLR4 pathway (Table 1). It can be seen from the above that apoptosis, oxidative stress, autophagy and inflammation are involved in the role of H_2_S regulation of ER stress in myocardial diseases. Therefore, clarifying the interaction between ER stress and the other physiological and pathological processes including apoptosis, oxidative stress, autophagy and inflammation in myocardial disease can well explain the mechanisms of H_2_S improving myocardial diseases. In addition, microRNAs have been reported to mediate H_2_S protection of myocardium (Hu et al., 2020; Su et al., 2021), however, the underlying mechanism is not fully understood. Hence, in the future, the role of microRNAs in H_2_S regulation of ER stress in cardiomyopathy is a subject worthy of study, which will provide a new target for the treatment of cardiomyopathy. The signal pathways involved in H_2_S regulation of ER stress in myocardial disease have rarely been studied. In addition to JAK/STAT pathway and TLR4 pathway, whether there are other signaling pathways participating in H_2_S regulation of ER stress in myocardial disease requires further research. Furthermre, some acute myocardial diseases, such as SIMD, need high concentration of H_2_S in a short time to improve the effect. However, there is still a lack of agents that can efficiently release H_2_S in a short time in clinical research. Therefore, exploring new and efficient H_2_S release agents will promote the treatment of myocardial diseases with H_2_S-related drugs.

**TABLE 1:** The role of hydrogen sulfide (H_2_S) regulation of endoplasmic reticulum stress in myocardial diseases.

The type of myocardial diseases	The role of hydrogen sulfide (H_2_S) regulation of endoplasmic reticulum stress	Experimental model	References
diabetes cardiomyopathy (DCM)	exogenous H_2_S improves DCM by inhibiting ER stress through reducing ROS/oxidative stress	rat model of DCM	Yang et al. (2016)
DCM	exogenous H_2_S may inhibit myocardial apoptosis through suppressing ER stress	rat model of DCM	Li et al. (2016)
DCM	exogenous H_2_S may ameliorate DCM through ROS/ER stress/autophagy/apoptosis by inhibiting JAK/STAT signaling pathway	rat model of DCM	Liu et al. (2018)
DCM	endogenous H_2_S was involved in DCM, and exogenous H_2_S ameliorated DCM by inhibiting ER stress	rat/AC 16 cells model of DCM	Guo et al. (2017)
ischemic/hypoxic myocardial disease	endogenous H_2_S protects against chronic intermittent hypoxia (CIH)-induced myocardial injury by reducing myocardial apoptosis, oxidative stress and ER stress	rat model of CIH	Zhou et al. (2018)
ischemic/hypoxic myocardial disease	exogenous H_2_S improved myocardial I/R injury through inhibiting I/R-induced ER stress and apoptosis of cardiomyocyte. and enhancing the proliferation, migration, and invasion of cardiomyocytes inhibited by I/R by upregulating miR-133a expression rats	H9C2 cells model of hypoxia-reoxygenation (H/R)	Ren et al. (2019)
**a**cute myocardial infarction (AMI)	exogenous H_2_S ameliorates myocardial remodeling after AMI by suppresseing cardiomyocyte apoptosis through regulating ER stress-autophagy axis	rat/H9C2 16 cells model of AMI	Li et al. (2020)
sepsis-induced myocardial dysfunction (SIMD)	endogenous H_2_S deficiency contributed to SIMD, and exogenous H_2_S improves LPS-induced SIMD through suppressing inflammation and ER stress by inhibiting TLR4 pathway	mice model of SIMD	Chen et al. (2021b)
